# A rare case of membrane pupillary-block glaucoma in a phakic eye with uveitis

**DOI:** 10.1186/s12886-025-04191-9

**Published:** 2025-07-01

**Authors:** Chiharu Iwahashi, Masahiko Fukuda, Shotaro Makita, Aya Takahashi, Tomoki Kurihara, Koji Sugioka, Shunji Kusaka

**Affiliations:** 1 Department of Ophthalmology, Kindai University Faculty of Medicine, 377-2, Ohnohigasi, Osakasayama, 589-8511 Osaka Japan; 2 Department of Ophthalmology, Kindai University Nara Hospital, Ikoma, Japan; 3 Department of Ophthalmology, Kyorin University School of Medicine, Tokyo, Japan

**Keywords:** Anterior segment imaging, Cataract surgery, Membrane, Pupillary-block glaucoma, Uveitis, Vitrectomy

## Abstract

**Background:**

Membrane pupillary-block glaucoma is a rare condition typically associated with fibrin formation due to postoperative inflammation following cataract surgery and vitrectomy.

**Case presentation:**

A 55-year-old man with a history of Stevens–Johnson syndrome and anterior uveitis presented with decreased vision in his right eye. Examination revealed corneal epitheliopathy, anterior chamber cells, and cataract. Increased oral steroids resolved the ocular inflammation, but one month later, he developed severe ocular pain in the right eye. Examination revealed best-corrected visual acuity (BCVA) of light perception, and intraocular pressure (IOP) was markedly elevated at 49 mmHg in the right eye. Anterior segment optical coherence tomography (AS-OCT) confirmed a shallow anterior chamber and a membrane across the pupil without adhesion to the lens, leading to the diagnosis of membrane pupillary-block glaucoma. AS-OCT was helpful in differentiating this condition from iris bombe. Surgical interventions, including membrane perforation, cataract extraction, and anterior vitrectomy, successfully relieved the pupillary block. Postoperatively, IOP decreased to 15 mmHg, and decimal BCVA improved to 0.4.

**Conclusion:**

This is the first reported case of membrane pupillary-block glaucoma in a phakic eye with uveitis, though it is typically reported postoperatively. AS-OCT is an invaluable diagnostic tool, which may enable prompt surgical intervention and lead to favorable outcomes.

## Background

Membrane pupillary-block glaucoma is a rare condition typically associated with fibrin formation, peripheral angle closure, and intraocular pressure (IOP) elevation following cataract surgery and vitrectomy [1,2,3,4]. Here, we present a unique case of membrane pupillary-block glaucoma in a phakic eye complicated by uveitis and successfully treated with cataract surgery and anterior vitrectomy.

## Case presentation

A 55-year-old male with obstructive bronchitis, on oral prednisolone 2.5 mg/day, presented to an ophthalmology clinic with decreased vision in the right eye. He had a history of Stevens–Johnson syndrome at age 33. He had no history of uveitis. He was diagnosed with a posterior subcapsular cataract in the right eye and was scheduled for cataract surgery; however, the procedure was aborted when continuous curvilinear capsulorrhexis forceps failed to reach the anterior capsule due to an unexplained transparent membrane in the anterior chamber. Subsequently, mild anterior segment inflammation of the right eye persisted and was treated with steroid eye drops.

**Fig. 1:**
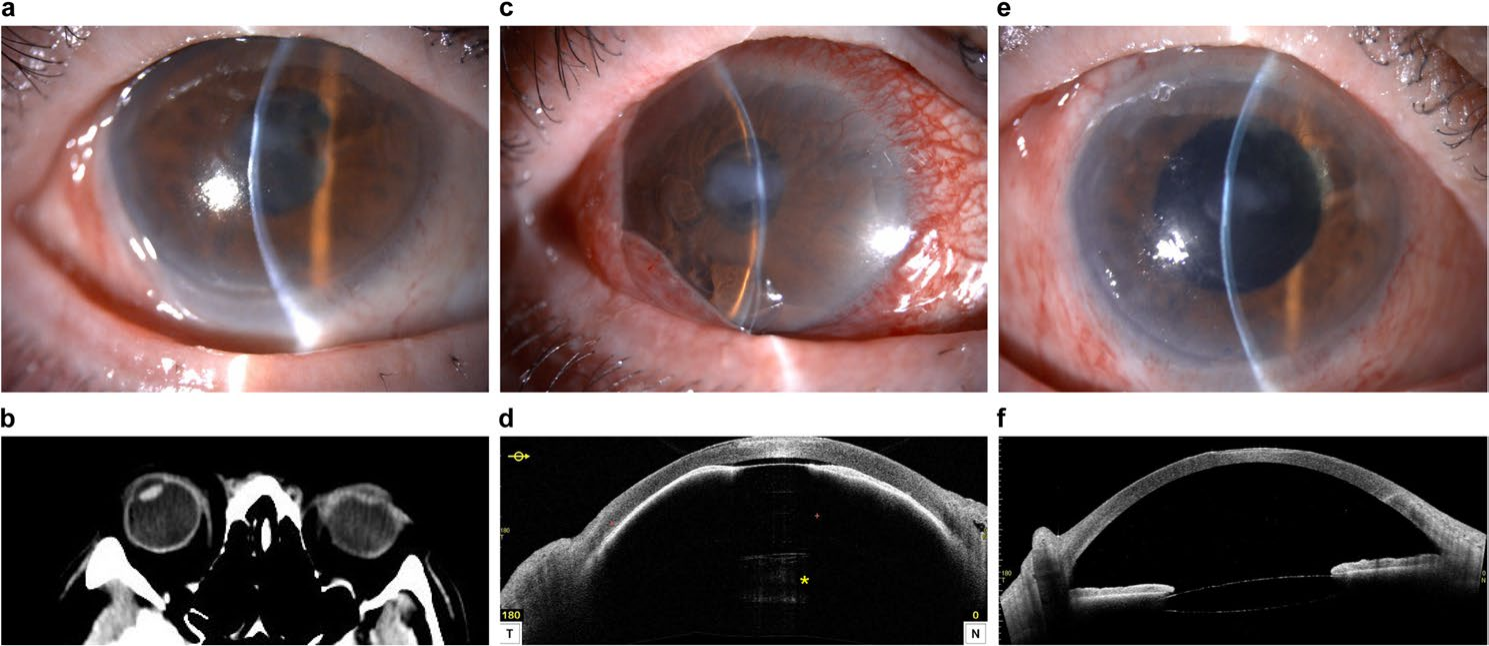
Pre- and Post-operative clinical findings of membrane pupillary-block glaucoma in a phakic eye. **A**. Slit-lamp examination at the initial visit to our hospital: a deep anterior chamber was observed. **B**. Computed tomography (CT) image taken in the emergency room one month after the initial visit showed that the cataractous lens was in its original position. **C**. Slit-lamp photograph taken on the same day as (**B**) showed an extremely shallow anterior chamber and marked conjunctival hyperemia. The pupillary zone details were obscured due to corneal opacity. **D**. Anterior segment optical coherence tomography (AS-OCT) image taken on the same day as (**B**) revealed an extremely shallow anterior chamber and the presence of a pupillary membrane. No lens displacement was observed (*). **E**. Slit-lamp photograph one month postoperatively showed a deep anterior chamber. **F**. AS-OCT image taken on the same day as (**E**) also showed a deep anterior chamber postoperatively

Eight months later, he was referred to the Department of Ophthalmology at Kindai University Hospital for anterior uveitis refractory to steroid eye drops. On examination, best-corrected visual acuity (BCVA) was hand motion in the right eye and 1.0 in the left. IOP was 8 mmHg in the right eye and 13 mmHg in the left. Slit-lamp examination of the right eye revealed a deep anterior chamber, anterior chamber cells (1+), corneal epitheliopathy, and cataract (Fig. 1A). There were no abnormal findings in the left eye. Increased oral steroids resolved the ocular inflammation.

One month later, he presented to the emergency department with severe headache and right eye pain. Brain computed tomography (CT) scans were unremarkable (Fig. 1B), and he was referred to the ophthalmology department for further evaluation. Examination revealed BCVA of light perception in the right eye and 1.0 in the left. IOP was markedly elevated at 49 mmHg in the right eye and normal at 9 mmHg in the left. Slit-lamp examination showed a shallow anterior chamber and severe corneal edema in the right eye (Fig. 1C). Anterior segment optical coherence tomography (AS-OCT) (CASIA2, Tomey Corp, Nagoya, Japan) confirmed a very shallow anterior chamber and a membrane across the pupil. The cataractous lens was in its original location, but there was a large clear space between the cataract and iris, with no physical contact between the two structures (Fig. 1D), which was also confirmed on the brain CT scan (Fig. 1B). Given these findings, a diagnosis of membrane pupillary-block glaucoma was made.

The patient underwent cataract surgery and anterior vitrectomy under retrobulbar anesthesia. During the procedure, the membrane across the pupil was perforated using a 25-gauge needle, and an iris retractor was used to dilate the pupil. This was followed by phacoemulsification cataract surgery and intraocular lens implantation.

On postoperative day 1, the anterior chamber was deep, and the IOP had normalized to 15 mmHg, yet corneal edema persisted. Oral prednisolone was started at 20 mg/day and tapered gradually to treat the postoperative inflammation. By postoperative week 1, corneal clarity had improved, and the anterior chamber remained deep (Fig. 1E and F). One month postoperatively, the patient’s BCVA improved to 0.4 in the right eye.

## Discussion

We reported a rare case of membrane pupillary-block glaucoma in a phakic eye with uveitis. The complete occlusion of the pupil by a fibrin membrane, with consequent peripheral angle closure and IOP elevation, is termed fibrin membrane pupillary-block glaucoma ¹ ² Postoperative fibrin membrane pupillary-block syndrome has been previously reported following pars plana vitrectomy and cataract surgery. However, in this case, pupillary-block glaucoma occurred in a phakic eye.

**Fig. 2:**
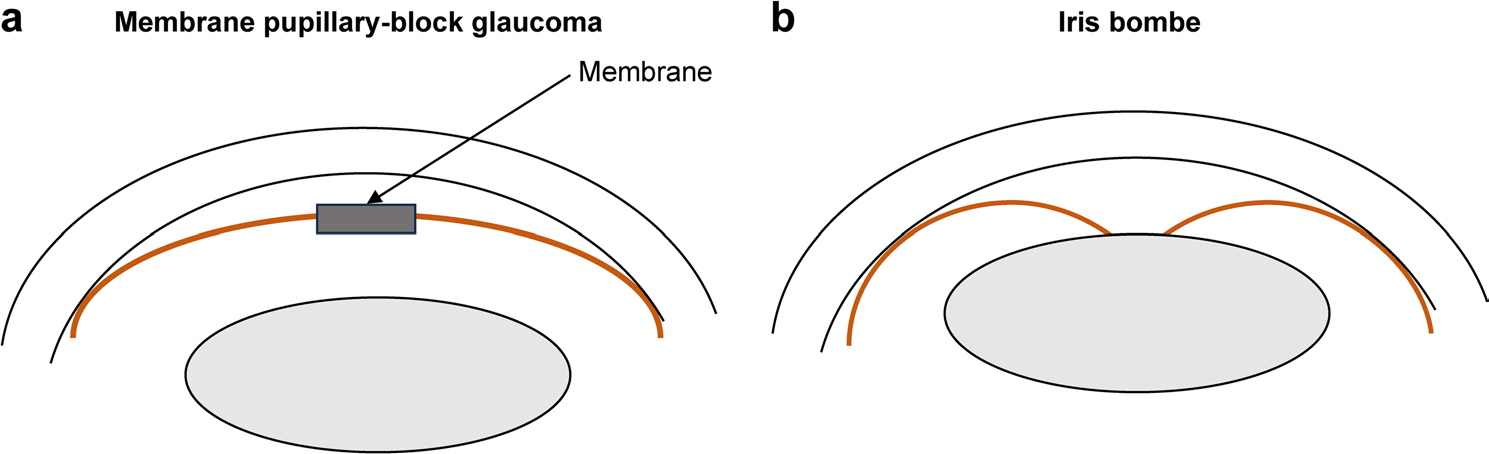
Schematic representation of AS-OCT findings in membrane pupillary-block glaucoma (**A**) and iris bombe (**B**). Both conditions present with a shallow anterior chamber; however, in membrane pupillary-block glaucoma, the iris does not touch the lens. In contrast, iris bombe is characterized by iris-lens adhesion with anterior iris bowing

Conventional slit-lamp examination is challenging with corneal edema, which obscure the lens position. In this case, the lens position was determined using findings from brain CT scans and AS-OCT. Although brain CT was performed in the emergency room due to address the patient’s severe headache, CT imaging involves radiation exposure and is not recommended as a routine diagnostic tool. In contrast, AS-OCT provides clear visualization of the anterior segment, as previously reported.⁴ Iris bombe in uveitic eyes is a unique acute angle-closure pathology, and differentiation from iris bombe secondary to uveitis was necessary. AS-OCT findings in iris bombe are characterized by anterior iris bowing and adhesion between the lens and iris,⁷ which were absent in this case, leading to a diagnosis of membrane pupillary-block glaucoma (Fig. 2).

Herein, poor mydriasis, potentially caused by the presence of membrane and inflammation, was observed at the initial visit. Pupil dilation improved after membrane removal, suggesting that the membrane contributed to restricted pupillary movement. Intraoperative use of an iris retractor facilitated further dilation. Irreversible mydriasis was not observed postoperatively. The patient’s limited visual recovery, with improvement only to 0.4, is presumably because corneal epithelial damage was associated with Stevens–Johnson syndrome. Although a definitive relationship cannot be established, Stevens-Johnson syndrome may also be relevant to the present condition. Chronic elevation of inflammatory cytokines in the aqueous humor has been reported in ocular surface diseases, including Stevens-Johnson syndrome [5], which may have influenced the development of uveitis.

Treatment options for fibrin membrane pupillary-block include laser peripheral iridotomy,¹ intraocular tissue plasminogen activator (tPA) injection, neodymium YAG (Nd: YAG) laser iridotomy,² and Nd: YAG fibrin membranotomy.³ [6] Herein, the very shallow anterior chamber made laser treatment and tPA injection technically difficult and posed potential risks to the corneal endothelium. Thus, the patient was treated with cataract surgery and vitrectomy, which successfully reduced IOP and improved visual acuity. Khor et al. reported four cases of postoperative fibrin pupillary-block glaucoma.³ Among these, two cases improved with Nd: YAG laser treatment of the membrane, one with topical and systemic glaucoma medications and topical prednisolone acetate, and another with membranectomy in the operating room. In this case, although membrane perforation alone may have sufficed to lower the IOP, additional surgical interventions ensured complete resolution.

The cellular composition of the membrane in this case remains unclear, but two possibilities are considered: fibrin caused by uveitis or a congenital membrane. Histological examination was not feasible because the membrane was too small to excise. If uncontrolled uveitis caused the membrane formation, it would typically result in posterior synechiae between the iris and lens, leading to iris bombe [7]. However, the iris configuration observed on AS-OCT differed from that of typical iris bombe [8]. The membrane may have been congenital, as it was observed at the beginning of the aborted cataract surgery at the referring hospital, and it may have adhered to the iris due to intraocular inflammation. To our knowledge, no similar cases have been reported, leaving its etiology undetermined.

The long-term risks of prolonged postoperative inflammation, which may require additional immunosuppressive treatment, must be considered. There is also a risk of membrane reformation following surgery, potentially necessitating further surgical interventions. These factors should be carefully monitored during the postoperative period to optimize long-term management and prevent complications.

We demonstrated that membrane pupillary-block glaucoma can develop in a phakic eye, although all previous reports of fibrin membrane pupillary-block syndrome occurred postoperatively. Prompt diagnosis and surgical intervention can lead to favorable outcomes. Further research and case documentation are needed to better understand this rare condition.

## Data Availability

No datasets were generated or analysed during the current study.

## Abbreviations

AS-OCT: Anterior segment optical coherence tomography
CT: Computed tomography
IOP: Intraocular pressure
Nd: YAG: Neodymium YAG
tPA: Tissue plasminogen activator
